# Implementing an Internet-Delivered Skin Cancer Genetic Testing Intervention to Improve Sun Protection Behavior in a Diverse Population: Protocol for a Randomized Controlled Trial

**DOI:** 10.2196/resprot.7158

**Published:** 2017-04-25

**Authors:** Jennifer L Hay, Marianne Berwick, Kate Zielaskowski, Kirsten AM White, Vivian M Rodríguez, Erika Robers, Dolores D Guest, Andrew Sussman, Yvonne Talamantes, Matthew R Schwartz, Jennie Greb, Jessica Bigney, Kimberly A Kaphingst, Keith Hunley, David B Buller

**Affiliations:** ^1^ Memorial Sloan Kettering Cancer Center Department of Psychiatry & Behavioral Sciences New York, NY United States; ^2^ University of New Mexico Albuquerque, NM United States; ^3^ Center for Mind+Body Health Charlottesville, VA United States; ^4^ University of Utah Salt Lake City, UT United States; ^5^ Klein Buendel, Inc. Golden, CO United States

**Keywords:** genetic testing, primary care, online health education, melanoma prevention, skin cancer risk, genetic risk communication

## Abstract

**Background:**

Limited translational genomic research currently exists to guide the availability, comprehension, and appropriate use of personalized genomics in diverse general population subgroups. Melanoma skin cancers are preventable, curable, common in the general population, and disproportionately increasing in Hispanics.

**Objective:**

Variants in the *melanocortin-1 receptor* (*MC1R*) gene are present in approximately 50% of the population, are major factors in determining sun sensitivity, and confer a 2-to-3-fold increase in melanoma risk in the general population, even in populations with darker skin. Therefore, feedback regarding *MC1R* risk status may raise risk awareness and protective behavior in the general population.

**Methods:**

We are conducting a randomized controlled trial examining Internet presentation of the risks and benefits of personalized genomic testing for *MC1R* gene variants that are associated with increased melanoma risk. We will enroll a total of 885 participants (462 participants are currently enrolled), who will be randomized 6:1 to personalized genomic testing for melanoma risk versus waiting list control. Control participants will be offered testing after outcome assessments. Participants will be balanced across self-reported Hispanic versus non-Hispanic ethnicity (n=750 in personalized genomic testing for melanoma risk arm; n=135 in control arm), and will be recruited from a general population cohort in Albuquerque, New Mexico, which is subject to year-round sun exposure. Baseline surveys will be completed in-person with study staff and follow-up measures will be completed via telephone.

**Results:**

Aim 1 of the trial will examine the personal utility of personalized genomic testing for melanoma risk in terms of short-term (3-month) sun protection and skin screening behaviors, family and physician communication, and melanoma threat and control beliefs (ie, putative mediators of behavior change). We will also examine potential unintended consequences of testing among those who receive average-risk personalized genomic testing for melanoma risk findings, and examine predictors of sun protection at 3 months as the outcome. These findings will be used to develop messages for groups that receive average-risk feedback. Aim 2 will compare rates of test consideration in Hispanics versus non-Hispanics, including consideration of testing pros and cons and registration of a decision to either accept or decline testing. Aim 3 will examine personalized genomic testing for melanoma risk feedback comprehension, recall, satisfaction, and cancer-related distress in those who undergo testing, and whether these outcomes differ by ethnicity (Hispanic vs non-Hispanic), or sociocultural or demographic factors. Final outcome data collection is anticipated to be complete by October 2017, at which point data analysis will commence.

**Conclusions:**

This study has important implications for personalized genomics in the context of melanoma risk, and may be broadly applicable as a model for delivery of personalized genomic feedback for other health conditions.

## Introduction

Melanoma is a rapidly increasing and preventable cancer in the general population. Melanoma incidence rates have increased more rapidly than any other cancer in the past several decades [1,2]. Melanoma accounts for 70% of skin cancer deaths each year [3], and is currently the fourth most common cancer among men and sixth most common among women, both in the Unites States [3] and in New Mexico [4]. Among Hispanics, disproportionate increases in melanoma (particularly thicker tumors with poorer prognoses) have been documented in states with high levels of year-round sun exposure, such as California and Florida [5-9]. For example, in 2010 Rouhani and colleagues [5] compared data from the Florida Cancer Data System (FCDS) with national incidence rates from the Surveillance, Epidemiology, and End Results (SEER) Program. Male Hispanics from the FCDS had a 20% higher incidence rate of melanoma between 1992 and 2004, relative to SEER. Nationally, incidence rates continue to rise among people of lower socioeconomic status and among older men [9-11]. In nonwhites, melanoma results in greater morbidity and mortality due to the disease often being identified at later stages, and because of low physician and patient awareness that melanomas occur in these populations [5,8,12-15]. By 2060, Hispanics will comprise 29% of the US population, further increasing the public health significance of melanoma in Hispanics [16].

Ultraviolet radiation delivered via sunlight is the predominant modifiable cause of melanoma, with approximately 65-90% of melanomas caused by ultraviolet radiation [17-19]. As such, melanoma risk reduction recommendations include daily sun protection, such as sun exposure avoidance, use of hats and clothing, and use of sunscreen [20]. However, most individuals do not use sunscreen, wear protective clothing, or seek shade on a regular basis [21], and in the United States, large general population surveys show that approximately 35% of the population uses sunscreen consistently [20,22,23]. This behavior extends to Hispanics of varying skin types [24,25], and Hispanics in the United States have high sunburn rates [26].

Personalized genomic testing for melanoma risk may promote risk awareness and risk reduction in the general population. Variants of the *melanocortin-1 receptor* gene (*MC1R*) confer moderate melanoma and basal cell cancer risks in the general population [27]. This gene is located on the long arm of chromosome 16 and is related to cutaneous pigmentation (eg, fair skin, red hair) [28-37]. A great deal of accumulated evidence, including systematic analyses of candidate genes, genome wide-association studies, and a recent meta-analysis of 12 melanoma case-control studies involving 6000 individuals [38], has identified nine risk-increasing variants for melanoma with odds ratios ranging from 1.42 (95% CI 1.09-1.85) to 2.45 (95% CI 1.32-4.55) [39].

Importantly, variation in *MC1R* is associated with melanoma risk after adjustment for hair color and skin type [32-34,40-42]. As such, *MC1R* predicts melanoma risk in African-American [43], Spanish [44], and Mediterranean populations [34], with at least one study indicating that *MC1R* may confer greater risk on individuals with darker skin, compared to those with lighter skin [45]. Across Hispanic and non-Hispanic populations, approximately 50% of individuals have at least one risk variant [40,45]. This frequency is consistent across Europe [46]. Hispanics in Albuquerque, New Mexico have substantial Spanish ancestry [47,48], so we expect to find the frequency of at least one risk variant to be 50% across Hispanic and non-Hispanic study participants [44].

The translation of personalized genomics into real-world general population application is necessary [49] but understudied [50]. The sequencing of the human genome [51] and the isolation of high-risk mutations in tumor suppressor genes has led to the rapid development of clinically useful genetic testing strategies for various uncommon hereditary cancer syndromes. Psychosocial research has highlighted predictors and outcomes of genetic testing in high-risk families who present in specialized clinics and receive extensive genetic counseling [52,53], and is increasingly addressing the needs of diverse, high-risk individuals and families [54,55]. However, since most research has been conducted in the context of familial disease, it is not clear how the general population will respond to personalized genomics. A 2016 report from the National Academy of Sciences has highlighted the pressing need to address access issues in genomic medicine [56]. Despite this need, for-profit companies are already marketing and offering genetic testing directly to consumers [57-60]. This model has largely bypassed behavioral research that could ensure broad utility and reach of this technology through diverse populations, arguing for the time-sensitive need to develop an empirical basis to maximize the benefits and minimize the harms of genomic feedback, even as evidence for specific gene variants and panels inevitably shifts over time [59].

Communication and health behavior theories inform the anticipated impact of personalized genomic testing for melanoma risk. We propose that the personal utility of personalized genomic testing for melanoma risk can be best understood via enhanced communication regarding skin cancer risk with physicians and family, and individual belief processes (ie, arousal of health threat and threat control beliefs). Communication with family and physicians concerning skin cancer risk might be especially important among some individuals, if family opinions are prioritized within a collectivist culture and value system [61]. Protection Motivation Theory [62,63] proposes that individual beliefs, including heightened illness threat (beliefs about severity, susceptibility) and heightened risk information about skin cancer, lead to protective health behaviors when control beliefs are high. Control beliefs involve confidence to perform the behavior (self-efficacy) and confidence in the effectiveness of the behavior (response-efficacy). This study tests the role of personalized genomic testing for melanoma risk in influencing control and illness threat beliefs and communication, which are proposed mediators of behavioral outcomes.

We will examine the reach (defined as consideration of the pros and cons of testing and registration of test decision) of a feasible, generalizable channel with high dissemination potential: the Internet. We will also compare factors (Hispanic ethnicity, health literacy, health system distrust, sociocultural factors) that may differentially impact reach. Over the past decade, the rapid pace of discovery of risk-influencing genes and the use of the Internet as an important source of health information have evolved in parallel. However, the use of the Internet for health information drops sharply and directly with literacy levels, so we will assess health literacy as a moderator of reach in this study [64]. To date, uptake of Internet direct-to-consumer personalized genomic testing has generally been concentrated among white, highly-educated, and health-literate consumers [65]. This disproportionate access is a clear media justice issue, as the continuation of these trends [65-67] could widen health knowledge gaps [68] and the *digital divide* [69,70] in underserved populations, in the context of personalized genomics.

**Figure 1:**
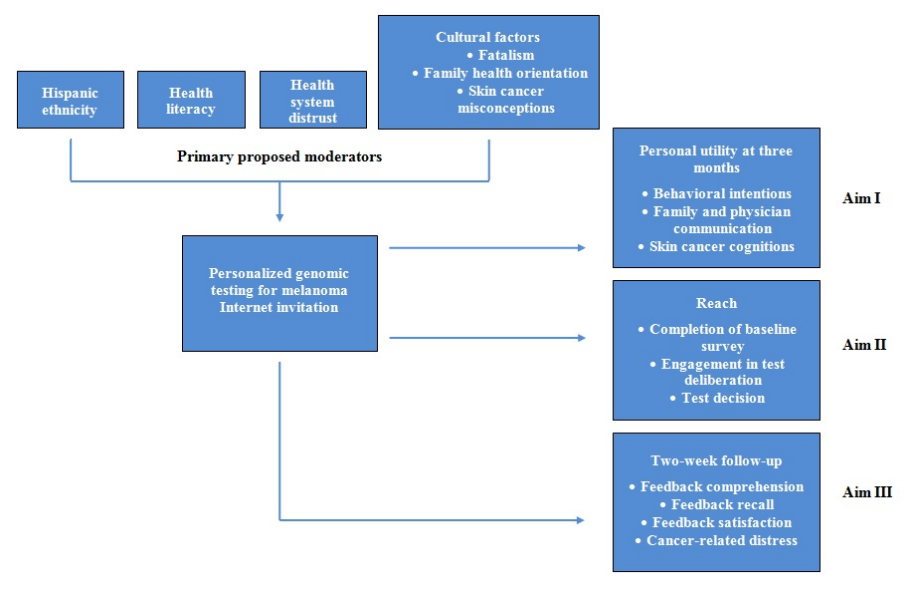
Conceptual model.

Based on these findings, prior work in primary care populations [71,72], as well as research examining barriers to participation among minority individuals in general cancer prevention trials [73,74], we anticipate that Hispanics may be less easily reached by personalized genomic testing for melanoma risk [75]. However, the Internet gives engaged individuals a direct method of accessing health information on a breadth of topics, and represents one of the most frequent reasons that individuals consult the Internet. A 2012 Pew Research Center Survey found that most general Internet users (66% of Hispanics; 73% of non-Hispanic whites) used the Internet to find health information [76]. A recent study indicated that Hispanics are highly receptive to online cancer information [77]. In the case of personalized genomics, the Internet could provide needed privacy for individuals to consider the benefits and drawbacks of testing. Indeed, for-profit companies have attempted to capitalize on this potential, and this may lead to disproportionate utilization among those who distrust (and may seek to bypass) the health system. Therefore, distrust of the health system, found to be highly relevant in minorities [78,79], may differentially impact reach. Finally, Hispanic sociocultural factors that are known to influence cancer prevention and screening activities [80], including cancer fatalism [81,82], an orientation to health that prioritizes the family over the individual [83], and specific misperceptions about skin cancer that are more common in Hispanics than non-Hispanics [15,84], may help us examine reasons for differential personalized genomic testing for melanoma risk reach. Examining these factors will help explain differences in reach by ethnic group, and thus provide critical direction in future personalized genomic testing for melanoma risk modifications for broad dissemination (see Figure 1).

### Specific Aims

This randomized controlled trial, “SOMBRA: Skin health Online for Melanoma: Better Risk Assessment” examines Internet presentation of the risks and benefits of personalized genomic testing for melanoma risk versus wait-list controls who are not offered testing. The study will compare personal utility and reach in a general population of English- or Spanish-speaking cohort in Albuquerque, New Mexico, which experiences year-round sun exposure.

In Aim 1, we will examine the personal utility of personalized genomic testing for melanoma risk in terms of short-term (3 months after testing) sun protection and skin screening (ie, behaviors), communication, and melanoma threat and control beliefs (ie, putative mediators of behavior change). Guided by Protection Motivation Theory [62], we hypothesize that behaviors and putative mediators will be higher in those who test, compared to those who decline testing or wait-list controls. Given that an important challenge of personal genomics involves the potential for those who receive *negative* genetic feedback to increase risky behaviors [85], we will also examine this potential unintended consequence of testing. To do so, we will conduct a subgroup analysis among those who receive average-risk personalized genomic testing for melanoma risk findings, and examine sun protection at 3 months as the outcome. Predictors will include baseline melanoma threat and control beliefs, melanoma risk factors, and demographics. These findings may be used in future studies to develop messages for groups that receive average-risk feedback, which accounts for large segments of those tested for moderate risk susceptibility factors across many diseases.

In Aim 2, we will examine differential reach of personalized genomic testing for melanoma risk between Hispanics and non-Hispanics, and potential explanations for any differential reach. Additional assessments of reach include baseline survey completion and the decision to pursue personalized genomic testing for melanoma risk testing. For the reasons listed above, we hypothesize that those who self-identify as Hispanic will show reduced reach, and that this reduction will in fact be the product of differences between Hispanics and non-Hispanics in relation to health literacy, health system distrust, and sociocultural factors [80], including cancer fatalism [81], family health orientation [83], and skin cancer misperceptions [15,84]. These results will inform future personalized genomic testing for melanoma risk modifications for Hispanics.

Finally, in Aim 3, among those who undergo testing we will examine test comprehension, recall and satisfaction, and cancer-related distress two weeks after test receipt. We will also examine whether these outcomes differ by ethnicity (Hispanic vs non-Hispanic) health literacy, health system distrust, sociocultural, or demographic factors.

## Methods

### Website Development and Usability Testing

Klein Buendel, Inc., a company specializing in health education programs and multimedia products in chronic disease prevention and control, provided the Web-based computer interface for the personalized genomic testing for melanoma risk education modules and testing invitation. Dr. David Buller (a study coinvestigator) is the Klein Buendel Research Director and an expert in skin cancer communications strategies. The Multiplex Study led by the National Human Genome Research Institute developed an Internet website where participants could opt to undergo genomic testing and risk feedback for common diseases, including the *MC1R* gene for melanoma risk, that was highly comprehensible, accurately interpreted, and did not increase distress in a primary care population [86,87]. We adapted these materials for our current study, and the modules are: (1) *What genetic testing can and cannot tell you*, (2) *Skin cancer and genes*, (3) *Your rights if you take part in genetic research*, and (4) *Your decision to be tested or not*. The website retains each of the four feasible Multiplex Study educational modules, with comprehension questions contained inside a website interface. The interface includes help files, navigation devices, and data collection code. The interface was tested and beta-tested by data professionals at Klein Buendel for stability and accuracy, and is hosted on secure data servers at Klein Buendel. Participants randomized to the personalized genomic testing for melanoma risk study arm view these materials via the Internet. To assess website usability, we conducted semistructured interviews (n=9) with English-speaking (n=8) and Spanish-speaking (n=1) primary care patients at 1209 Clinic, a University of New Mexico (UNM) General Internal Medicine Clinic that represents the primary recruitment site for the study. Any issue raised by at least one participant or study team member was evaluated for revision. Overall, participants found the website usable with 18 problems identified (eg, web pages with too much text, confusing wording, and unclear instructions). The research team developed solutions for these problems, which were confirmed with Klein Buendel before implementation.

### Spanish Translation and Cognitive Interviews

We followed published guidance for translation and cognitive interviewing drawn from *Translation, Review and Adjudication, Pretesting, and Documentation* procedures [88,89]. First, the Memorial Sloan Kettering Cancer Center Linguistic and Cultural Competence Team (led by Mr. Javier Gonzalez and Dr. Francesca Gany, coinvestigators) within the Immigrant Health and Cancer Disparities Service provided Spanish translations and certificates of authenticity of all study materials, including: study invitation flyer, baseline survey, personalized genomic testing for melanoma risk Internet educational modules and corresponding knowledge surveys, buccal cell sample provision instructions, risk feedback comprehension assessment, 3-month telephone outcome survey, and consent forms. We internally reviewed the documents and provided fine-tuning.

Next, we conducted semistructured cognitive interviews (n=28) to assess the comprehension and acceptability of translated study materials with our target population (primary care Spanish-speaking Hispanic patients at 1209 Clinic at UNM) stratified across gender and education levels (≥high school, <high school). Bilingual interviewers administered only a portion of the materials to each participant to reduce patient burden and maximize completion with at least two patients viewing every item. If any issues were raised by at least one participant, a research assistant or the investigator panel (multidisciplinary team composed of experts in qualitative data analysis, linguistic translation, health and genetic literacy, and anthropology) evaluated the item for revision and labeled it as a problem [90]. Procedural details and results are reported elsewhere [91]. Most materials were comprehensible and acceptable, but 33 of 246 terms/concepts were not. These items were modified by the multidisciplinary team and retested. During this phase the team adopted the term *skin cancer* rather than *melanoma* in Internet and risk communication materials, due to the greater comprehensibility of this term in the translation and cognitive interviews.

### Randomized Controlled Trial

In our ongoing randomized controlled trial, our bilingual Research Study Assistants approach primary care patients in UNM General Internal Medicine clinics with invitation flyers (English and Spanish) and National Cancer Institute skin cancer information for diverse skin types (available in English and Spanish versions; “ *Anyone can get skin cancer* ”). Patients are eligible for the study if they have been registered in any UNM clinic for at least six months, assigned a primary care physician in the UNM system, are aged ≥18 years, and are fluent in English or Spanish. We originally limited recruitment to the 1209 Clinic, but expanded to other UNM clinics to boost recruitment rates. If patients are eligible and interested in study participation, they complete the Baseline Survey (including an informed consent form) in-clinic via a semiprivate space with a Research Study Assistant who enters their responses on a dedicated study computer (tablet or laptop computer with wireless Internet access). In prior preliminary studies with UNM primary care patients, we found high levels of receptivity to skin cancer genomic information, yet higher skin cancer misconceptions than in the general population, making this an appropriate study context [84].

If patients are eligible but not interested in participating, we assess reasons for study refusal and ask them to complete a one-minute Refuser Survey (skin cancer risk perceptions, interest in genomic technologies, and demographics). Based on the Multiplex Study [86,87], we expect a 30% baseline survey response rate, for a total sample size of 885. After completion of the Baseline Survey, participants receive US $15 for their time and effort, and either a referral to consider personalized genomic testing for melanoma risk through a secure website *or* wait-list control (randomized 1:6; balanced across Hispanic vs non-Hispanic ethnicity; n=135 in control arm, n=750 in personalized genomic testing for melanoma risk arm). Hispanic ethnicity will be recorded by self-report. Trial design and reporting will adhere to The Consolidated Standards for Reporting Trials Statement [92,93]. Patients will choose Spanish or English study materials, and we will record their preference.

After completing the baseline survey in-clinic, all participants randomized to consider personalized genomic testing for melanoma risk are given an introductory letter inviting them to log onto the study website at their earliest convenience (preferably within the next month) to read the four educational modules regarding personalized genomic testing for melanoma risk, and to answer a series of questions regarding comprehension of, and satisfaction with, the content of each module. In section 4, participants register a test decision. Participants are only able to register a test decision if they have already read and completed the questions in the educational modules. Those who complete these steps receive a US $5 gift card for each educational module completed, for a total of US $15 in gift cards. Registration of a test decision (yes vs no) is our primary assessment of reach in this study. We expect a minimum of 30% of participants who complete the Baseline Survey to register a test decision, and that this will reach 50% in some subgroups, including those with higher literacy, and non-Hispanic subgroups [94]. Additional assessments of reach include completion of the baseline survey and decision to pursue personalized genomic testing for melanoma risk testing (yes vs no). Those who register a decision to proceed with testing will receive deoxyribonucleic acid (DNA) buccal cell test kits which will allow them to provide a saliva sample for genetic testing, postage prepaid envelopes, and instructions for buccal cell collection. Participants can return their kits at any point. Genetic counseling sessions are available at the participants’ request. In accordance with The Multiplex Study [72], we anticipate that 50% of those who consider personalized genomic testing for melanoma risk will return a saliva sample for genetic testing. Testing will be conducted on samples that are received by the lab and results will be mailed within one month.

Genomic DNA will be isolated from buccal cells using Oragene (Ottawa, ON, Canada). The Oragene kit generally provides at least 110 micrograms of high quality DNA. Standard polymerase chain reaction will be used to amplify the 951-nucleotide *MC1R* coding region. All amplified products will be sequenced on an ABI Prism 3100 (Applied Biosystems, Foster City, CA) using BigDye Terminators (Applied Biosystems) according to manufacturer’s specifications. Sequencing primers are:

5’-TCGTCTTCAGCACTCTCTTC-3’

5’-TTTAAGGCCAAAGCCCTGGT-3’

5’-AACCTGCACTCACCCATGTA-3’

5’-CTGCAGGTGATCACGTCAAT-3’

*MC1R* chromatograms will be read with the aid of Sequencher software version 4.05 (Gene Codes Corp., Ann Arbor, MI) and/or SeqScape software versions 1.0 to 2.1.1 (Applied Biosystems). These data will be read independently by two reviewers. This procedure is standard and has been used in most recent studies examining DNA isolation. We will sequence the coding region of *MC1R* in participants’ germline DNA and identify all variants. *MC1R* variants are typically classified as their risk for red hair (“R” variant) and low risk (“r” variant). The *MC1R* variants identified will be characterized as: (1) nonsynonymous or synonymous, (2) coding or noncoding, and (3) *MC1R* variants that are strongly associated with melanoma risk and represented as V60L, D84E, V92M, R142H, R151C, I155T, R160W, R163Q, and D294H; the nine variants that are associated with melanoma, regardless of skin type [41]. Genotype definitions used in these analyses are adapted from work exploring the association between *MC1R* variants and melanoma risk [39]. We estimate that 50% of participants will receive findings concerning at least one *MC1R* risk variant [40,45].

To provide risk feedback, molecular genotypes are combined into two categories: (1) *average-risk feedback*, the presence of no *MC1R* risk alleles associated with risk of melanoma; or (2) *higher risk feedback*, the presence of at least one *MC1R* risk allele, which is associated with risk of developing melanoma (range of 1.47 [95% CI 1.17-1.84] to 2.74 [95% CI 1.53-4.89]). We employ state-of-the-art methods of risk communication used with high comprehension in the Multiplex Study [86,87]. These materials include verbal and picture displays of risk information [95,96], given that people often neglect base rates for a disease and have difficulty understanding joint probabilities and shifting denominators [97]. We will also provide written information to clarify the *bottom line* information, given that individuals tend to rely on the gist of the information [97,98].

All participants who undergo personalized genomic testing for melanoma risk will receive a follow-up call two weeks after results are mailed to them, to assess result comprehension and potential distress (Risk Feedback Comprehension Assessment). All participants who complete this assessment will receive a US $5 gift card. Based on prior literature documenting low levels of distress in individuals undergoing genetic testing for high-risk mutations [99], and those found to carry *Cyclin-Dependent Kinase Inhibitor 2A* mutations indicating high melanoma risk [100], we expect low levels of distress regarding personalized genomic testing for melanoma risk feedback. Those who report high distress will be referred for follow-up by Clinic Director Dr. Jessica Bigney, who addresses distress issues in the UNM clinic.

**Figure 2:**
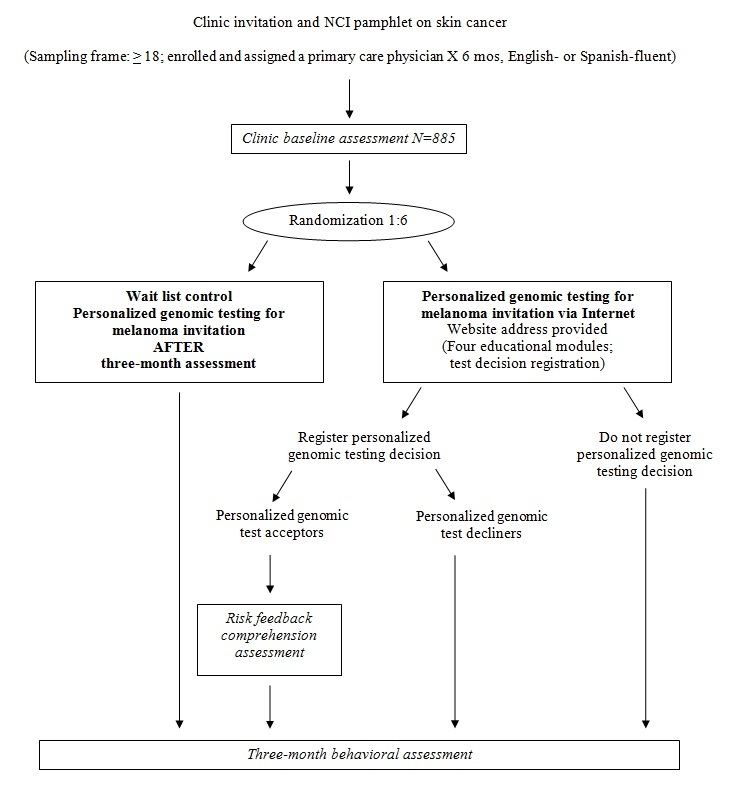
Study flow. PGT-M: personalized genomic testing for melanoma.

All participants who complete Baseline Assessments (whether tested or not) are contacted by telephone after 3 months. Participants who complete the follow-up survey receive a US $15 gift card. See Figure 2 for study flow. Measures are outlined in Multimedia Appendix 1.

### Data Analysis

We will examine four aspects of data quality and distributional assumptions: (1) data skewness, kurtosis, and parametric assumptions; (2) intention-to-treat principles; (3) missing data considerations; and (4) control of potentially inflated type-1 errors due to multiple statistical tests. We assume that up to 20% of the respondents will be unreachable at follow-up; missing assessments may be amenable to imputation by several techniques [101,102].

We will use a series of regression analyses to examine the personal utility of personalized genomic testing for melanoma risk. For these analyses, the dependent variables will be: short-term (3-month) sun protection and skin screening (ie, behaviors); communication; and skin cancer threat and control beliefs (ie, putative mediators of behavior change) [62,63,103]. Given guidelines regarding the importance of consistent use of sunscreen [3], patient-reported sunscreen use frequency will be dichotomized (*frequent* or more vs *sometimes* or less) to indicate consistent versus inconsistent use. Our expected approximate sample size for this analysis will be 708, assuming 20% attrition of our original 885 participants. Personalized genomic testing for melanoma risk uptake status will be categorized into three groups: (1) those who undergo personalized genomic testing for melanoma risk (*acceptors*), (2) those who decline personalized genomic testing for melanoma risk (*decliners*), and (3) those who are not offered personalized genomic testing for melanoma risk (*controls*). A logistic regression model will be used for sun protection outcomes that are dichotomous (eg, consistent use vs inconsistent use) as a function of personalized genomic testing for melanoma risk uptake. The baseline outcome assessments will be entered as covariates to provide control for ceiling and floor effects. Primary moderators will be considered, including ethnicity (Hispanic vs non-Hispanic), health literacy, health system distrust, sociocultural factors (cancer fatalism, family health orientation, skin cancer misconceptions), and high-risk versus average-risk personalized genomic testing for melanoma risk feedback, as well as demographics and skin cancer risk factors. Average ultraviolet index over the 3-month assessment time period will be considered as a covariate to provide control over seasonal variations in Albuquerque. The hypothesis is supported if there is a significant difference in sunscreen use between personalized genomic testing for melanoma risk acceptors and personalized genomic testing for melanoma risk decliners or controls, such that acceptors show greater sunscreen adherence.

Decreased sun protection may be an unintended consequence of testing among those who receive average-risk personalized genomic testing for melanoma risk feedback [85]. To examine this possibility, we will conduct a subgroup analysis among those who receive average-risk personalized genomic testing for melanoma risk feedback, and examine sun protection outcomes at 3 months as the outcome. Predictors will include baseline skin cancer threat and control beliefs, melanoma risk factors, demographics, and sociocultural factors (health literacy and health system distrust, cancer fatalism, family health orientation, and skin cancer misconceptions). Given the expected sample size for the analysis (n=60; participants who undergo testing and receive average-risk personalized genomic testing for melanoma risk feedback), we will use univariate analyses to guide predictor selection. Nonparametric statistics will be considered when appropriate to guard against violations of parametric assumptions in this restricted sample.

We will examine differential reach of personalized genomic testing for melanoma risk across Hispanics and non-Hispanics, and potential explanations for any differential reach. Reach is defined as registration of a personalized genomic testing for melanoma risk test decision, either accepting or declining testing (dichotomous outcome; test decision or no test decision). Additional assessments of reach include baseline survey completion and decision to pursue personalized genomic testing for melanoma risk testing. Only participants randomized to the personalized genomic testing for melanoma risk arm–those who are offered personalized genomic testing for melanoma risk–will be included in this analysis. Our sample size for this analysis will be 600, assuming 20% attrition of the original 750 who are offered personalized genomic testing for melanoma risk. We aim to offer putative explanations, such as differences in health literacy or skin cancer misconceptions concerning *why* Hispanics offered personalized genomic testing for melanoma risk may be less likely to register a personalized genomic testing for melanoma risk decision. This analysis will involve a moderation framework [104,105], such that reduced reach in Hispanics is moderated by one or more third variables (eg, skin cancer misconceptions). We will use a logistic regression modeling framework to address Aim 2. A standard requirement in moderation analysis [105] entails two sequential statistical findings: (1) there should first be a statistically significant Hispanic effect in Model 1; and (2) after adjusting for the moderator of interest in Model 2, the previously significant main Hispanic effect will no longer be significant. This approach may be applied to a variable coding *Hispanic* (yes vs no) and a moderator variable such as *skin cancer misconceptions*. This analysis will be applied to other putative explanations of why Hispanics might be less likely to register a personalized genomic testing for melanoma risk decision.

Among personalized genomic testing for melanoma risk test acceptors, we will examine (two weeks after test result receipt) test comprehension, recall, satisfaction, and distress. Our sample size for this analysis will be approximately 90, given that we expect to reach 80% (90/114) of those who undergo personalized genomic testing for melanoma risk testing, and thus receive personalized genomic testing for melanoma risk feedback. Based on the Multiplex Study [86,87], we anticipate that personalized genomic testing for melanoma risk feedback will be read by at least 80% of participants who undergo personalized genomic testing, and that at least 80% will correctly recall and accurately interpret their results. We anticipate that most participants (>95%) will report low levels of distress, including nervousness, testing regret, fear, and confusion. We will examine these outcomes using bivariate statistics, and examine differences across ethnicity, health literacy, health system distrust, and sociocultural factors.

### Statistical Power

Regarding personal utility in Aim 1, we hypothesize that there will be higher rates of sunscreen use in personalized genomic testing for melanoma risk test acceptors, compared to personalized genomic testing for melanoma risk decliners or controls. We predict that those who accept personalized genomic testing for melanoma risk will have higher levels of sunscreen use (65% regular sunscreen use, consistent with rates of sunscreen use in those with melanoma risk factors [106]), compared to 35% sunscreen use in decliners or controls (consistent with rates of sunscreen use in the general population [20,22,23]). We estimated the statistical power in personal utility between personalized genomic testing for melanoma risk accepters compared to decliners or controls. The comparison between a 65% versus 35% difference in sunscreen use was carried out using Cohen’s method [107]. An estimated 65% versus 35% contrast translates to an arcsine-transformed effect size index of 0.61 [107], which yields a statistical power of 99.7% in a hypothesis test of these two proportions between personalized genomic testing for melanoma risk acceptors (an estimated n=90 after 20% attrition) and decliners (n=509 after 20% attrition), at a two-sided test with a tail probability of 0.01 (lower than the conventional 0.05 tail probability to reserve power for subset analyses).

Regarding the outcome of reach in Aim 2, we hypothesize that Hispanics will show reduced reach, but that differences in health literacy, health system distrust, and sociocultural factors (cancer fatalism, family health orientation, skin cancer misconceptions) will explain these findings. This method involves testing a moderation relationship in a logistic regression model. To estimate the statistical power, we ran 400 simulated logistic regression models, assuming Hispanics at 50% of the sample, and that within the Hispanic group high skin cancer misconceptions would be associated with a 0.38 odds ratio in personalized genomic testing for melanoma risk test registration; high skin cancer misconceptions would be associated with lower personalized genomic testing for melanoma risk registration. When converted to Cohen d, this 0.38 odds ratio translates to an effect size of -0.54, which Cohen considers a *medium* effect size. Based on the Multiplex Study [86], we estimate a minimum of 30% of participants will register a personalized genomic testing for melanoma risk decision among Hispanics, and a higher reach of 50% among non-Hispanics. This 30% versus 50% difference translates to a Cohen effect size of 0.50 [107]. We estimate 81% statistical power to detect a medium effect size at a conventional two-sided type-1 error rate of 5% for a moderator analysis.

In sum, we have adequate power for personal utility and reach to (1) ensure adequate representation of individuals with low health literacy, (2) ensure robust protection against missing data, and (3) ensure sufficient statistical power to detect moderation. If the effect size is larger than estimated, such as a 30% versus 80% difference and a Cohen effect size of 0.65, then we would have power to spare for additional comparisons.

## Results

To date, 462 participants have been recruited to the study (203/462, 43.9% Hispanic; 222/462, 48.1% non-Hispanic white; 356/462, 77.1% female; mean age=54) and randomized 1:6 to usual care or the personalized genomic testing for melanoma risk offer. Final outcome data collection is anticipated to be complete by October 2017, at which point data analyses will commence.

## Discussion

This study is one of the first population-based efforts to widen the reach of personal genomics in *real-world* settings. Along with other work examining ways to maximize the use of the Internet to bring emerging technologies [108] (including genomics [109]) to the general population, our research will use genomic information to raise melanoma risk awareness and prevention and control behaviors for this rapidly increasing cancer, which is extremely hard to treat when diagnosed beyond stage 1. We use a rigorous randomized controlled trial design, which increases the rigor of the proposal by comparing those who undergo personalized genomic testing for melanoma risk testing to: (1) those who have declined, and (2) those who have not been offered personalized genomic testing for melanoma risk testing. We use an established and feasible approach to Web-based communication regarding skin cancer genetic testing, and measure utility and reach using a real-world approach by which the general population may realistically access it (Internet personalized genomic testing for melanoma risk invitation). We have identified a highly diverse population for assessment in a geographical location that is exposed to year-round sun exposure.

The current proposal will examine potential unintended consequences of *actual* genetic testing, by directly examining those who receive personalized genomic testing for melanoma average-risk results to identify predictors of behavior change in this group. Examination of this question will have important implications for personalized genomics in the context of melanoma risk, and will be broadly applicable as a model for delivery of personalized genomic feedback for other health conditions.

Study limitations include the fact that we do not assess participants for Internet literacy, and do not include detailed assessments regarding context of sun exposure (occupational, recreational). Finally, we did not employ blinding of study condition among study staff.

### Conclusions

Our findings will have important implications for personalized genomics in the context of melanoma risk, and will be broadly applicable as a model for delivery of personalized genomic feedback for other conditions in this population. We plan future work to expand personalized genomic testing for melanoma risk to include other melanoma risk and protective markers, and to expand risk stratification to multiple levels as the literature on genetic factors in skin cancer unfolds.

## Appendix

Multimedia Appendix 1 Study measures.

Multimedia Appendix 2 Peer-review reports from National Cancer Institute grant.

Multimedia Appendix 3 CONSORT publication form.

## Abbreviations

DNA: deoxyribonucleic acid
FCDS: Florida Cancer Data System
MC1R: melanocortin-1 receptor
SEER: Surveillance, Epidemiology, and End Results
SOMBRA: Skin health Online for Melanoma: Better Risk Assessment
UNM: University of New Mexico
