# Supplementary material for: Implementing an Internet-Delivered Skin Cancer Genetic Testing Intervention to Improve Sun Protection Behavior in a Diverse Population: Protocol for a Randomized Controlled Trial
Source: JMIR Res Protoc. 2017 Apr 25;6(4):e52. doi: 10.2196/resprot.7158 (PMC5424125; doi:10.2196/resprot.7158)
Supplement: Multimedia Appendix 1 [file resprot_v6i4e52_app1.pdf]

| Construct/Measure | Prior work/psychometrics | Assess point | Hypotheses |
|---|---|---|---|
| Sun protection and skin screening (Sunscreen, shade seeking, clothing and hat usage) on separate 5-point scales (never-always); health provider screening (no/yes). | Item reliability >80% [110]. | B, 3-month | For Aim I Personal Utility Sun protection will be higher for those accepting versus declining personalized genomic testing for melanoma, or controls. |
| Family/physician communication. Frequency, history, content of communication with physicians, family, and friends regarding skin cancer risk, interest in genetic information; 4-point scales (not at all-a lot). | Items used with high comprehensibility in population-based studies [111-115]. | B, 3-month, and Refuser survey (interest in genetic information only) | For Aim I Personal Utility Communication will be higher for those accepting versus declining personalized genomic testing for melanoma or controls. |
| Perceived skin cancer threat beliefs [116]. Includes assessment of risk perceptions (verbal, percent likelihood, comparison) on separate scales (5 to 10-point) from low-high, with "don't know" option [117]); worry, assessed with 4 4-point items (never-all the time); severity, 4 5-point items (strongly disagree-strongly agree). We also assess Cancer Risk Beliefs [121, 122]: 13 items assessing cognitive causation and negative affect related to cancer risk beliefs (4-pont scales, strongly disagree-strongly agree).<br>Perceived skin cancer control beliefs [116]. Includes skin cancer prevention self-efficacy (7 items, on separate 4-point scales; not at all- | Widely used perceived risk items drawn from prior health behavior research [118], Lerman's cancer worry scale [52] and perceived cancer severity [119]. Control belief items [120] predicting uptake of cancer prevention behaviors; adapted for skin cancer prevention [123]. | B, 3-month, and Refuser survey (risk perception question only) | For Aim I, putative mediators of Personal Utility Three-month threat and control beliefs will be higher for those accepting versus declining personalized genomic testing for melanoma or controls. |

| | | | |
|---|---|---|---|
| extremely capable), skin cancer prevention response-efficacy (7 items, on separate 4-point scales; not at all to extremely important). | | | |
| Hispanic ethnicity | For initial sampling frame (50%) self-reported Hispanic ethnicity. | B | For Aim II Reach will be higher for Non-Hispanics versus Hispanics. |
| Health Literacy includes 3 items: level of confidence in filling out medical forms independently, frequency of needed assistance reading hospital materials, and frequency of problems learning about medical conditions because of difficulty reading hospital materials (5-point scales, none of the time-all the time). | Single Item Literacy Screener Items [124, 125] are feasible in primary care populations, with more limited respondent burden compared to TOFHLA or REALM; Area under Receiver Operating Characteristic Curve analyses indicate good sensitivity for diverse literacy levels. | B | For Aim II Reach will be higher among those with higher versus lower health literacy (effect modifier). |
| Health System Distrust includes 9 items assessing two domains; values and competence distrust in 5-point scales (strongly agree-strongly disagree). | The Health System Distrust Scale [126] is validated in primary care against established physician trust scales; reliability overall (0.83), Values (0.73); Competence (0.77). | B | For Aim II Reach will be influenced by health system distrust; direction not proposed (effect modifier). |
| Sociocultural factors includes a 15-item assessment of Cancer Fatalism on 2-point scales (agree/disagree), a 4-item assessment of Family Health Orientation: social influences on learning more about health, social influences on doing more about health, how motivated they are to do what important others want them to do, how much their health choices affect others (7-point scales, strongly disagree- strongly agree), 10-item assessing Skin Cancer Misconceptions, | Cancer Fatalism predicts cancer prevention activities [81]; we use Powe Cancer Fatalism Scale [127] examining fear, pessimism, death inevitability, and predetermination beliefs. It is well-validated [81] and reliable (.88). Family Health Orientation assessed via social influences on health information seeking and behavior change from Multiplex [114], and Skin Cancer Misconceptions | B | For Aim II Reach will be higher in those who report less versus more cancer fatalism, more versus less family health orientation (family support for health), and among those with fewer versus more skin cancer misconceptions. |

| | | | |
|---|---|---|---|
| including preventability, treatability, and information overload about skin cancer (Agree/Disagree). | items from the Health Information National Trends Survey 2007 [119] and found to be lower among Hispanics [84]. | | |
| Knowledge and satisfaction with Internet personalized genomic testing for melanoma invitation modules, all on 7-point scales (strongly disagree-strongly agree). | Adopted from the NHGRI Multiplex Study [87]. | Embedded in personalized genomic testing for melanoma invitation | Treatment Fidelity assessments. |
| Test result comprehension includes recall, perceived clarity, interpretation and recall, believability, and test regret on closed, open ended scales. | Adopted from the NHGRI Multiplex Study [72, 86]. | Risk feedback comp. assessment | In Aim III, high comprehension of personalized genomic testing for melanoma feedback. |
| Cancer-related Distress is assessed through 7 items on separate 5-point scales (not at all-extremely). | Impact of Events Scale – Revised Intrusive thoughts subscale [129] is widely used with good internal and test–retest reliability; good ability to distinguish those with cancer distress [130, 131]. | Risk feedback comp. assessment; 3- month | In Aim III, low distress of personalized genomic testing for melanoma feedback. |
| Demographics (birth year, US nativity, survey language choice, gender, education, income, race/ethnicity); Internet availability. | Standard demographic questions; Internet availability [132]. | B, Refuser survey | Potential covariates for all Aims. |
| Melanoma risk factors (personal and family melanoma history; phenotype, sunburn hx). | Heavily used items from prior epidemiology research [133]. | B | Potential covariates for all Aims. |
