# Supplementary material for: Implementing an Internet-Delivered Skin Cancer Genetic Testing Intervention to Improve Sun Protection Behavior in a Diverse Population: Protocol for a Randomized Controlled Trial
Source: JMIR Res Protoc. 2017 Apr 25;6(4):e52. doi: 10.2196/resprot.7158 (PMC5424125; doi:10.2196/resprot.7158)
Supplement: Multimedia Appendix 2 [file resprot_v6i4e52_app2.pdf]

SUMMARY STATEMENT

(Privileged Communication)

PROGRAM CONTACT:
Heather Patrick
240.276.6795
heather.patrick@nih.gov

Release Date: 02/27/2014

Application Number: 1 R01 CA181241-01A1

Principal Investigators (Listed Alphabetically):
BERWICK, MARIANNE PHD
HAY, JENNIFER L PHD (Contact)

Applicant Organization: SLOAN-KETTERING INST CAN RES

Review Group: SEIR
Societal and Ethical Issues in Research Study Section

Meeting Date: 02/10/2014
Council: MAY 2014
Requested Start: 07/01/2014

RFA/PA: PA11-250
PCC: K7HP

Project Title: Personalized Genomic Testing for Melanoma: Maximizing Personal Utility and Reach
SRG Action: Impact Score: 25 Percentile: 12
Next Steps: Visit http://grants.nih.gov/grants/next_steps.htm
Human Subjects: 30-Human subjects involved - Certified, no SRG concerns
Animal Subjects: 10-No live vertebrate animals involved for competing appl.
Gender: 1A-Both genders, scientifically acceptable
Minority: 1A-Minorities and non-minorities, scientifically acceptable
Children: 3A-No children included, scientifically acceptable
Clinical Research - not NIH-defined Phase III Trial

Project Year	Direct Costs Requested	Estimated Total Cost
1	495,963	830,326
2	497,955	833,661
3	499,679	836,547
TOTAL	1,493,597	2,500,533

ADMINISTRATIVE BUDGET NOTE: The budget shown is the requested budget and has not been adjusted to reflect any recommendations made by reviewers. If an award is planned, the costs will be calculated by Institute grants management staff based on the recommendations outlined below in the COMMITTEE BUDGET RECOMMENDATIONS section.

1R01CA181241-01A1 HAY, JENNIFER

RESUME AND SUMMARY OF DISCUSSION: This project will evaluate the personal utility of personalized genomic testing for melanoma (PGT-M) risk among a diverse Hispanic and non-Hispanic population in New Mexico. Melanoma is an important public health problem and the incidence of disease continues to rise and the increase has been disproportionate and with a poorer prognosis among non-Caucasians. The investigators are an outstanding multi-disciplinary team with appropriate expertise for the project. The investigators have been very responsive to the previous review and have strengthened the project. Strengths of the project include the preliminary data, the RCT of comparing the PGT-M via internet versus a wait list control, the theoretical framework, recruitment and data collection, and measurement approaches. The project may be strengthened by recruiting for PGT-M test acceptors and then randomizing the group to either receive results immediately or later. This project from an outstanding group of investigators will improve our understanding of the impact of genomic testing in healthy individuals.

DESCRIPTION (provided by applicant): Currently little translational genomic research exists to guide the availability, comprehension, and appropriate uptake of personalized genomics in diverse, general population subgroups that stand to benefit from it in the coming years. The Multiplex Study led by the National Human Genome Research Institute (NHGRI) developed an Internet offer of genomic testing and risk feedback for common diseases, including the melanocortin receptor gene (MC1R) for melanoma risk, that was highly comprehensible, accurately interpreted, and did not increase distress in a primary care population. Melanoma skin cancers are preventable, curable, common in the general population, and disproportionately increasing in Hispanics. Higher risk variants in MC1R are present in about 50% of the population, interact with sun exposure, and confer 2-3 fold melanoma risk in the general population - even darker skin populations - thus feedback regarding MC1R risk status is a potential vehicle to raise risk awareness and protective behavior in the general population. We propose a randomized controlled trial examining Internet presentation of the risks and benefits of personalized genomic testing for melanoma (PGT-M) via MC1R testing (N=885, randomized 6:1 PGT-M versus waiting list control offered testing after outcome assessments, balanced across Hispanic versus Non-Hispanic ethnicity, n=750 in PGT-M arm; n=135 in control arm) comparing personal utility and reach in a general population cohort in Albuquerque New Mexico, where there is year-round sun exposure. Aim I will examine the personal utility of PGT-M in terms of short-term (three month) sun protection, skin screening (i.e., behaviors), communication, melanoma threat and control beliefs (i.e., putative mediators of behavior change). We hypothesize that behaviors and putative mediators will be higher in those who test compared to those who decline testing. Aim 1a will examine potential unintended consequences of testing among those who receive average risk PGT-M findings, examining predictors of sun protection at three months as the outcome. These findings will be used to develop messages for groups that receive average risk feedback. Aim II will compare rates of reach of PGT-M in Hispanic versus Non-Hispanics in terms of consideration of the pros and cons of testing and registration of PGT-M decision. We hypothesize that Hispanics will show reduced reach, but that levels of health literacy, health system distrust, and sociocultural factors (cancer fatalism, family health orientation, skin cancer misconceptions) will explain differences in reach between Hispanics and Non- Hispanics, and provide guidance for future PGT-M modifications for Hispanics. Aim III will examine PGT-M feedback comprehension, recall, satisfaction, and cancer-related distress in those who undergo testing, and whether these outcomes differ by ethnicity (Hispanic versus Non-Hispanic) or sociocultural or demographic factors. The current study will be the first to use the established Multiplex invitation for skin cancer genetic risk testing to examine behavioral outcomes, and the first to use Multiplex to engage a Hispanic population - neither was addressed in the original Multiplex Study. The study will have important implications for personalized genomics in the melanoma context, and will be broadly applicable as a model for delivery of personalized genomic feedback for other conditions, as well. PHS 398/2590 (Rev. 06/09) Page Continuation Format Page

PUBLIC HEALTH RELEVANCE: This is a randomized controlled trial examining personal utility (sun protection behavior change, communication, cognitions) and test reach (consideration of testing) in participants randomized to Internet approach to presenting the risks and benefits of personalized genomic testing for melanoma (PGT-M; via MC1R testing) vs. waiting list control (offered testing after 3-month outcome assessments) in a general population cohort in Albuquerque New Mexico that is 50% Hispanic, 50% Non-Hispanic. Examination of this question will have important implications for personalized genomics in the melanoma context, and will be broadly applicable as a model for delivery of personalized genomic feedback for other conditions, as well.

CRITIQUE 1:

Significance: 2
Investigator(s): 1
Innovation: 2
Approach: 4
Environment: 1

Overall Impact: This is a resubmission of a proposal to rigorously study the impact of personalized genetic testing for melanoma. The public health question is important in and of itself, and the question of whether personalized genetic testing can actually change behavior is a very important one at this time in the development and popularization of genomic technologies. The investigators have taken a grant that was already extremely well written and well-crafted and improved it, making the entire proposal more feasible and more relevant to “real life”.

1. Significance:

Strengths

- Melanoma is an important health care issue
- Findings from a study of this common risk marker may be applicable to other genetic risk markers
- The false reassurance condition explored in Aim 1a has been suggested as important but has not been rigorously studied.
- The effort to include Hispanic subpopulation continues to be a strength.

Weaknesses

- Even though DTC genomics has been set back by the recent FDA decision, the future of genomics is likely to be panels rather than single risk markers as proposed here. It is recognized that adding additional markers, even the modest number that were provided in the Multiplex study, as the intervention in a RCT, would be quite burdensome. It seems important to pause and ask whether providing a single risk marker like this will ever be done in the future, and if not, what is the relevance of this design to future health outcomes.
- Three month follow up in a relatively brief time to see a change that will be lasting.

2. Investigator(s):

Strengths

- Dr. Hay’s more limited experience well balanced by involved and experienced co-investigators.

Weaknesses

- None noted.

3. Innovation:

Strengths

- Real world RCT
- Internet approach

Weaknesses

- Patient reported outcomes may not be accurate or last beyond 3 months

4. Approach:

Strengths

- Well-designed, methodologically rigorous, statistically well considered trial design.
- Design is simplified and much improved for real-world feasibility and potential public health impact in comparison to the first proposal.

Weaknesses

- I doubt that the Multiplex response rate will be the same in the population proposed.
- The investigators did not address the concern about distinguishing “average risk” from “lower risk”. I remain concerned that this is mathematically incorrect, and therefore an incorrect message to designate one group as higher risk and the other group as average risk. Moreover, perhaps the most important question, whether or not those not at increased risk will avoid sunscreen, will be less likely to be found without this.
- Patient reported sunscreen use is likely subject to social desirability that may be triggered by the genetic information. A better control might be genetic information plus public health admonition vs. public health admonition alone.
- The design is mostly much improved, but I think the new design has a flaw in pitting wait-list controls against PGT-M test acceptors. The randomization will not be equivalent this way. They should recruit PGT-M test acceptors and THEN randomize that group to receive results immediately or later.

5. Environment:

Strengths

- Strong and supportive.

Weaknesses

- None.

Protections for Human Subjects:

Acceptable Risks and/or Adequate Protections

Data and Safety Monitoring Plan (Applicable for Clinical Trials Only):

Acceptable

Inclusion of Women, Minorities and Children:

G1A - Both Genders, Acceptable

M1A - Minority and Non-minority, Acceptable

C3A - No Children Included, Acceptable

Vertebrate Animals:

Not Applicable (No Vertebrate Animals)

Biohazards:

Not Applicable (No Biohazards)

Resource Sharing Plans:

Acceptable

Budget and Period of Support:

Recommend as Requested

CRITIQUE 2:

Significance: 2
Investigator(s): 1
Innovation: 2
Approach: 4
Environment: 1

Overall Impact: This is a revised application from an exceptional team of investigators that is highly responsive to the prior critique; as a result, this is a stronger application in many respects. The topic is novel and the approach builds on prior work of several team members. Melanoma is a growing public health problem; thus, the focus of this study is highly significant. The changes in study design since the last submission raise some questions about the potential uptake (or lack thereof) of an internet-based intervention, and how that might impact the overall findings and dissemination potential. However, in general, the strengths outweigh the weaknesses of this application.

1. Significance:

Strengths

- Melanoma is an important public health problem, as the incidence of disease continues to rise in the US population. Among Hispanics, this increase has been disproportionate and reflects the diagnosis of thicker tumors with poor prognosis, compounded with the greater possibility of later diagnosis and low provider awareness of risk among nonwhites.

Weaknesses

- The design is modeled after the prior Multiplex Study, however the methodology suggests potential limitations in regard to internet access which may pose limitations in this study.

2. Investigator(s):

Strengths

- Outstanding multidisciplinary team with exceptional and complementary expertise necessary to carry out this research.

Weaknesses

- None noted.

3. Innovation:

Strengths

- This study will build upon the work conducted previously in the Multiplex Study Intervention specifically by focusing on a primarily Hispanic general population sample in the southwestern US, and also by focusing on behavioral outcomes related to sun protection.

Weaknesses

- None noted.

4. Approach:

Strengths

- The investigators have been responsive to the prior critique: the study design has been modified to include two arms- the PGT-M provided via internet vs. a wait-list control who will be offered the intervention 3 months post-final assessment. Rationale for the revised approach is that the prior Multiplex study showed that delivering the information via the internet is an effective and feasible approach.
- The theoretical framework, recruitment and data collection, and measurement approaches are sound and well-described.
- Preliminary data presented in a paper that is in press supports the rationale for focusing on skin cancer prevention behaviors in Hispanics.

Weaknesses

- With the modification in study design to the two-arm study (PGT-M via internet vs. wait list control), this suggests that another indicator of reach may involve the mode of delivery itself; specifically, whether those in the PGT-M arm actually log on to the website and read the information. It would have been helpful for the investigators to provide preliminary data on uptake of intervention via internet in the target population. While the investigators cite the previous success of the Multiplex Study in using this mode of delivery for testing information as well as for indicating a testing decision, the potential uptake (as well as barriers to uptake) of this intervention in this population are unknown.
- In addition, the study design does not appear to deliberately track whether participants actively log on to the website. This is a potentially important intermediate step that could affect the primary outcomes. It is not clear whether such participants would be considered in the attrition estimates. The study design also does not appear indicate whether participants in the intervention arm are expected to log into the website within a certain time period after completion of the baseline assessment, and if failure to do so would constitute attrition.

- It would be helpful to know whether participants in the intervention arm will be provided access to a computer and the internet at the clinic to access the study website, if so desired.
- Intervention delivered by internet only is likely to exclude persons who do not typically access or feel comfortable using the internet, which may include those who are lower SES, low acculturated, older persons. This detracts from the dissemination potential if the intervention is successful.

5. Environment:

Strengths

- Excellent.

Weaknesses

- None noted.

Protections for Human Subjects:

Acceptable Risks and/or Adequate Protections

Inclusion of Women, Minorities and Children:

G1A - Both Genders, Acceptable

M1A - Minority and Non-minority, Acceptable

C3A - No Children Included, Acceptable

Vertebrate Animals:

Not Applicable (No Vertebrate Animals)

Biohazards:

Not Applicable (No Biohazards)

Resubmission:

- Very responsive to prior critique.

Budget and Period of Support:

Recommend as Requested

CRITIQUE 3:

Significance: 3
Investigator(s): 1
Innovation: 2
Approach: 2
Environment: 2

Overall Impact: This is an outstanding study and the investigators have addressed the reviewers' comments. The redesign to a randomized waitlist control of internet-based materials will provide important knowledge about healthily individuals and the Hispanic population about an important health issue. The investigative team is outstanding and the extension of the Multiplex platform effectively leverages a well-developed resource. However, the study may not have long-term clinical utility to improve the health of this population.

1. Significance:

Strengths

- The study is now focused on Internet based delivery and compares those who are tested and those who decline testing and those who are not offered testing. The focus on the web-based application is an improvement.
- The focus on Hispanic population is important

Weaknesses

- The application may overstate the potential utility of this information and even the moniker "personalized" genomic testing may overstate its value
- The long-term clinical utility of this approach will not be addressed in this three-month study.

2. Investigator(s):

Strengths

- The PIs have complete strengths and have assembled an outstanding team.

Weaknesses

- None noted.

3. Innovation:

Strengths

- Focus on healthy people
- Extrapolation of the multiplex to this context.

Weaknesses

- None noted.

4. Approach:

Strengths

- Thoughtful conceptual approach to address this question
- Emphasis on language and cultural issues by study team members
- Development of supplemental web based materials to augment the multiplex approach
- Decision to use a 3 month outcome
- Randomization prior to decisions about testing allows for more comparisons between groups.

Weaknesses

- The three month outcome is measurable but less clear how meaningful it will be as a pragmatic endpoint.
- This study will improve our understanding of psychological aspects of testing in healthy individuals but the rigor of the design and approach may limit its clinical impact on directly improving health.

5. Environment:

Strengths

- Well suited institution

Weaknesses

- None noted.

Protections for Human Subjects:

Acceptable Risks and/or Adequate Protections

Data and Safety Monitoring Plan (Applicable for Clinical Trials Only):

Acceptable

Inclusion of Women, Minorities and Children:

G1A - Both Genders, Acceptable

M1A - Minority and Non-minority, Acceptable

C3A - No Children Included, Acceptable

Vertebrate Animals:

Not Applicable (No Vertebrate Animals)

Biohazards:

Not Applicable (No Biohazards)

Resubmission:

- Very thoughtful consideration and response to the reviewer comments

Budget and Period of Support:

Recommend as Requested

THE FOLLOWING RESUME SECTIONS WERE PREPARED BY THE SCIENTIFIC REVIEW OFFICER TO SUMMARIZE THE OUTCOME OF DISCUSSIONS OF THE REVIEW COMMITTEE ON THE FOLLOWING ISSUES:

PROTECTION OF HUMAN SUBJECTS (Resume): ACCEPTABLE

INCLUSION OF WOMEN PLAN (Resume): ACCEPTABLE

INCLUSION OF MINORITIES PLAN (Resume): ACCEPTABLE

INCLUSION OF CHILDREN PLAN (Resume): ACCEPTABLE

COMMITTEE BUDGET RECOMMENDATIONS: The budget was recommended as requested.

NIH has modified its policy regarding the receipt of resubmissions (amended applications). See Guide Notice NOT-OD-10-080 at http://grants.nih.gov/grants/guide/notice-files/NOT-OD-10-080.html.
The impact/priority score is calculated after discussion of an application by averaging the overall scores (1-9) given by all voting reviewers on the committee and multiplying by 10. The criterion scores are submitted prior to the meeting by the individual reviewers assigned to an application, and are not discussed specifically at the review meeting or calculated into the overall impact score. Some applications also receive a percentile ranking. For details on the review process, see http://grants.nih.gov/grants/peer_review_process.htm#scoring.

MEETING ROSTER

Societal and Ethical Issues in Research Study Section
Population Sciences and Epidemiology Integrated Review Group
CENTER FOR SCIENTIFIC REVIEW
SEIR
February 10, 2014

CHAIRPERSON

WHITE, DOUGLAS B, MD
UPMC ENDOWED CHAIR FOR ETHICS IN CRITICAL CARE
ASSOCIATE PROFESSOR OF CRITICAL CARE MEDICINE
DIRECTOR, PROGRAM ON ETHICS AND DECISION MAKING
IN CRITICAL ILLNESS
UNIVERSITY OF PITTSBURGH
PITTSBURGH, PA 15261

MEMBERS

BAILEY, DONALD B JR, PHD *
DISTINGUISHED FELLOW, EARLY CHILDHOOD DEVELOPMENT
RESEARCH TRIANGLE INTERNATIONAL
RESEARCH TRIANGLE PARK, NC 27709

DOHAN, DANIEL P., PHD *
ASSOCIATE PROFESSOR IN RESIDENCE
INSTITUTE FOR HEALTH POLICY STUDIES
UNIVERSITY OF CALIFORNIA
SAN FRANCISCO, CA 94118

DUBOIS, JAMES M, DSC, PHD
PROFESSOR OF MEDICINE
DIVISION OF GENERAL MEDICAL SCIENCES
DIRECTOR, CENTER FOR CLINICAL RESEARCH ETHICS
INSTITUTE FOR CLINICAL AND TRANSLATIONAL SCIENCE
WASHINGTON UNIVERSITY IN ST. LOUIS
ST. LOUIS, MO 631101093

GREEN, ROBERT C, MPH, MD
ASSOCIATE PROFESSOR OF MEDICINE
ASSOCIATE DIRECTOR FOR RESEARCH
PARTNERS CENTER FOR PERSONALIZED GENETIC MEDICINE
BRIGHAM AND WOMEN'S HOSPITAL
HARVARD MEDICAL SCHOOL
BOSTON, MA 02115

HARRIS, TINA MARIA, PHD *
JOSIAH T MEIGS DISTINGUISHED TEACHING PROFESSOR
AFFILIATE, INSTITUTE OF AFRICAN AMERICAN STUDIES
AFFILIATE, QUALITATIVE INTEREST GROUP
DEPARTMENT OF COMMUNICATION STUDIES
UNIVERSITY OF GEORGIA
ATHENS, GA 30602

KIM, SCOTT Y, MD, PHD
SENIOR INVESTIGATOR, MEDICAL OFFICER
CLINICAL CENTER
DEPARTMENT OF BIOETHICS
NATIONAL INSTITUTES OF HEALTH
BETHESDA, MD 20892

LEE, SANDRA SOO-JIN, PHD
SENIOR RESEARCH SCHOLAR
CENTER FOR BIOMEDICAL ETHICS
STANFORD UNIVERSITY MEDICAL SCHOOL
STANFORD, CA 94305

MERZ, JON F, PHD, JD
ASSOCIATE PROFESSOR
DEPARTMENT OF MEDICAL ETHICS AND HEALTH POLICY
AND CENTER FOR BIOETHICS
PERELMAN SCHOOL OF MEDICINE
UNIVERSITY OF PENNSYLVANIA
PHILADELPHIA, PA 19104

PETERSON, SUSAN K, PHD *
ASSOCIATE PROFESSOR
DEPARTMENT OF BEHAVIORAL SCIENCE
M.D. ANDERSON CANCER CENTER
THE UNIVERSITY OF TEXAS
HOUSTON, TX 77030

WILFOND, BENJAMIN SIMON, MD *
DIRECTOR, TREUMAN KATZ CENTER FOR PEDIATRIC BIOETHICS
UNIVERSITY OF WASHINGTON SCHOOL OF MEDICINE
SEATTLE, WA 98101

MAIL REVIEWER(S)

MARCHANT, GARY E, PHD, JD
PROFESSOR OF LAW
SANDRA DAY O'CONNOR COLLEGE OF LAW
ARIZONA STATE UNIVERSITY
TEMPE, AZ 852877906

SCIENTIFIC REVIEW ADMINISTRATOR

HELMERS, KARIN F, PHD
SCIENTIFIC REVIEW OFFICER
CENTER FOR SCIENTIFIC REVIEW
NATIONAL INSTITUTES OF HEALTH
BETHESDA, MD 20892

GRANTS TECHNICAL ASSISTANT

BURGESS, ANGELA
EXTRAMURAL SUPPORT ASSISTANT
CENTER FOR SCIENTIFIC REVIEW
NATIONAL INSTITUTES OF HEALTH
BETHESDA, MD 20892

SUTERWALA, NISRIN
ADMINISTRATIVE ASSISTANT
DIVISION OF EXTRAMURAL ACTIVITIES SUPPORT
CENTER FOR SCIENTIFIC REVIEW
NATIONAL INSTITUTES OF HEALTH
BETHESDA, MD 20892

* Temporary Member. For grant applications, temporary members may participate in the entire meeting or may review only selected applications as needed.

Consultants are required to absent themselves from the room during the review of any application if their presence would constitute or appear to constitute a conflict of interest.
